# Bisphosphonates-induced Atypical Subtrochanteric Fracture Femur: A Case Report

**DOI:** 10.7759/cureus.2937

**Published:** 2018-07-06

**Authors:** Siddhart Yadav, Vikram Khanna

**Affiliations:** 1 Orthopaedics, Apollo Hospital, Mumbai, IND; 2 Orthopaedics, Ranjana Hospital, Allahabad, IND

**Keywords:** orthopaedics, nonunion, subtrochanteric fracture, bisphosphonates

## Abstract

A 71-year-old female came to our institute with a subtrochanteric left femur fracture following a fall from her bed. It was a low-energy trauma, and the X-rays were suggestive of an atypical fracture following bisphosphonate therapy for five years. The fracture was fixed with intramedullary nailing following which she was started on partial weight-bearing walking after three weeks. The fracture went on to a nonunion; after one year, the fracture site was opened and bone grafting with bone marrow injection, along with augmented plating, was done. The fracture showed signs of union three months postoperatively. Fractures associated with bisphosphonate therapy may be associated with delayed union or nonunion and should be explained to the patient.

## Introduction

Over the past 20 years, bisphosphonates have become the mainstay for the management of osteoporosis [1]. They increase bone mineral density and, in turn, decrease the chances of fragility fractures [2]. This is achieved by decreasing bone resorption. However, long-term treatment by bisphosphonates is associated with loss of the capability of bone turnover, which may decrease the capacity of the bone to remodel [1]. Hence, long-term treatment may lead to a decrease in bone strength due to various microtraumas, and in turn, decrease the toughness of the bone and cause the bones to become brittle [3]. This has been very well demonstrated in recent articles, which have shown the high incidence of subtrochanteric or femur shaft fractures in patients with osteoporosis on long-term bisphosphonate treatment [4].

Fractures associated with long-term bisphosphonate use are different from osteoporosis fractures of the proximal femur as they are caused by low-energy trauma and they have typical radiological features like thickening of the medial cortex with a fracture of the lateral cortex [4]. Many times, it is also associated with prodromal symptoms even before the fractures occur. These symptoms include thigh pain which may be present months before the actual fracture occurs [5]. Although recently, there has been an increase in the number of such cases, no general consensus has been reached regarding the method of management of such fractures [1]. Here, we discuss a case where an insufficiency fracture was seen in the subtrochanteric region, and it was primarily managed with proximal femoral nailing. However, it later went into nonunion and was managed by additional plating with bone grafting and bone marrow concentrate after one year.

## Case presentation

**Figure 1:**
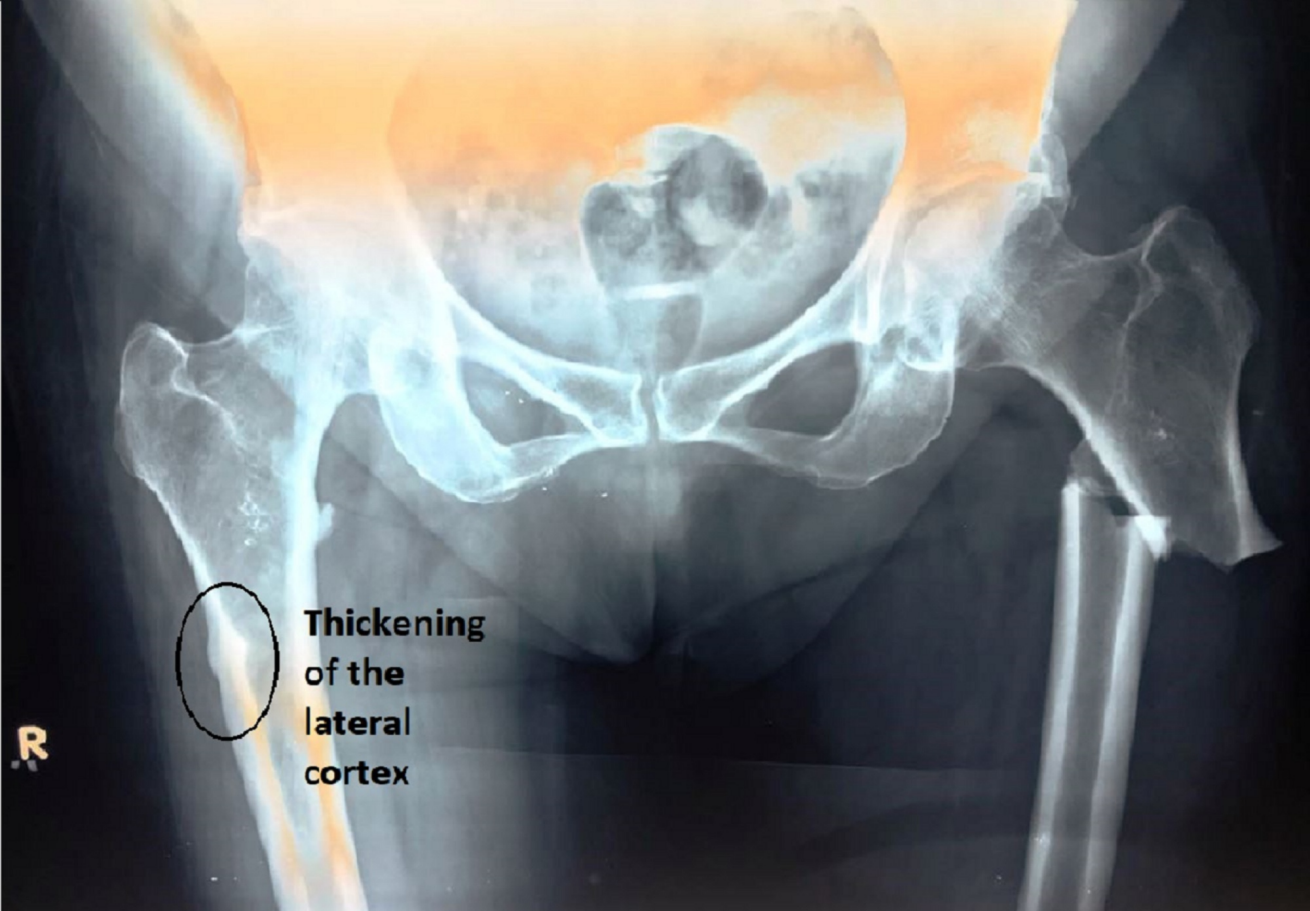
X-ray pelvis with hips antero-posterior view showing subtrochanteric fracture of the left femur with localized thickening of the lateral cortex of right femur subtrochanteric region.

A 71-year-old female came to our institute after falling down from her bed in September 2016. She sustained an injury on her left thigh. She also gave a history of taking bisphosphonates (alendronate, 70 mg weekly) continuously for the past five years. On examination, she was unable to walk or put any weight on her left leg. Her left leg was in complete external rotation, and she was unable to do active straight leg raises (SLR). With the help of an X-ray, a subtrochanteric fracture of the left femur was diagnosed (Figure 1). The right thigh X-ray also showed thickening of the lateral cortex, which was indicative of the changes occurring due to the bisphosphonates. The patient was a known case of controlled diabetes mellitus and hypertension.

**Figure 2:**
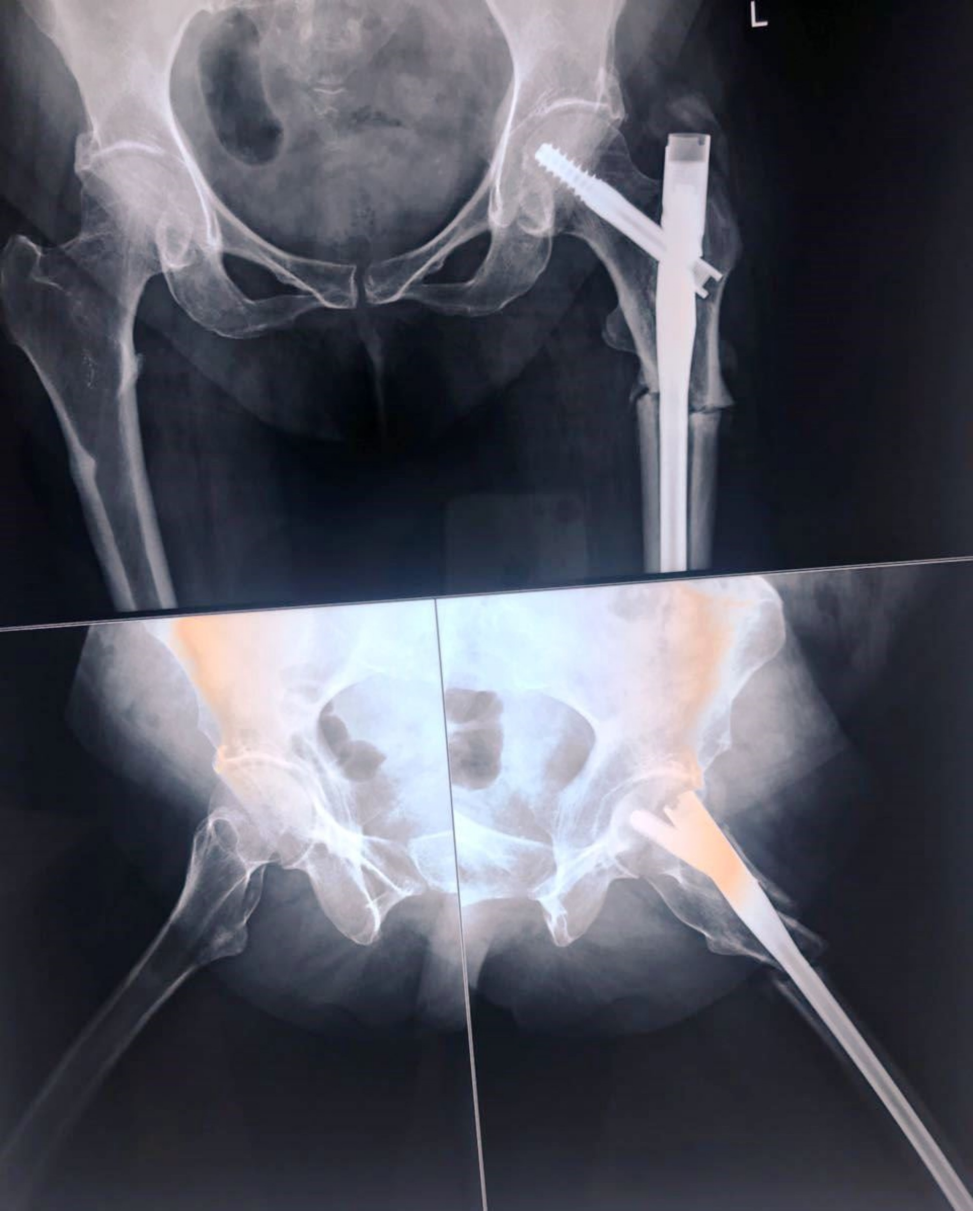
Immediate postoperative X-ray showing the intramedullary nail in situ.

After the necessary preoperative investigations, the patient was taken up for fixation with close proximal femoral interlock nailing of the trochanteric fracture of the left femur (Figure 2). As there was no breach in the cortex seen on the right side, no operative intervention was planned. After fixation, the patient was started on partial weight-bearing walking after three weeks. After reaching the required serum calcium, serum Vitamin D, and serum parathyroid hormone (PTH) levels, the patient was started on teriparatide, 8 IU subcutaneous daily injections, along with elemental calcium, 500 mg, and Vitamin D, 60,000 IU weekly, for six months.

**Figure 3:**
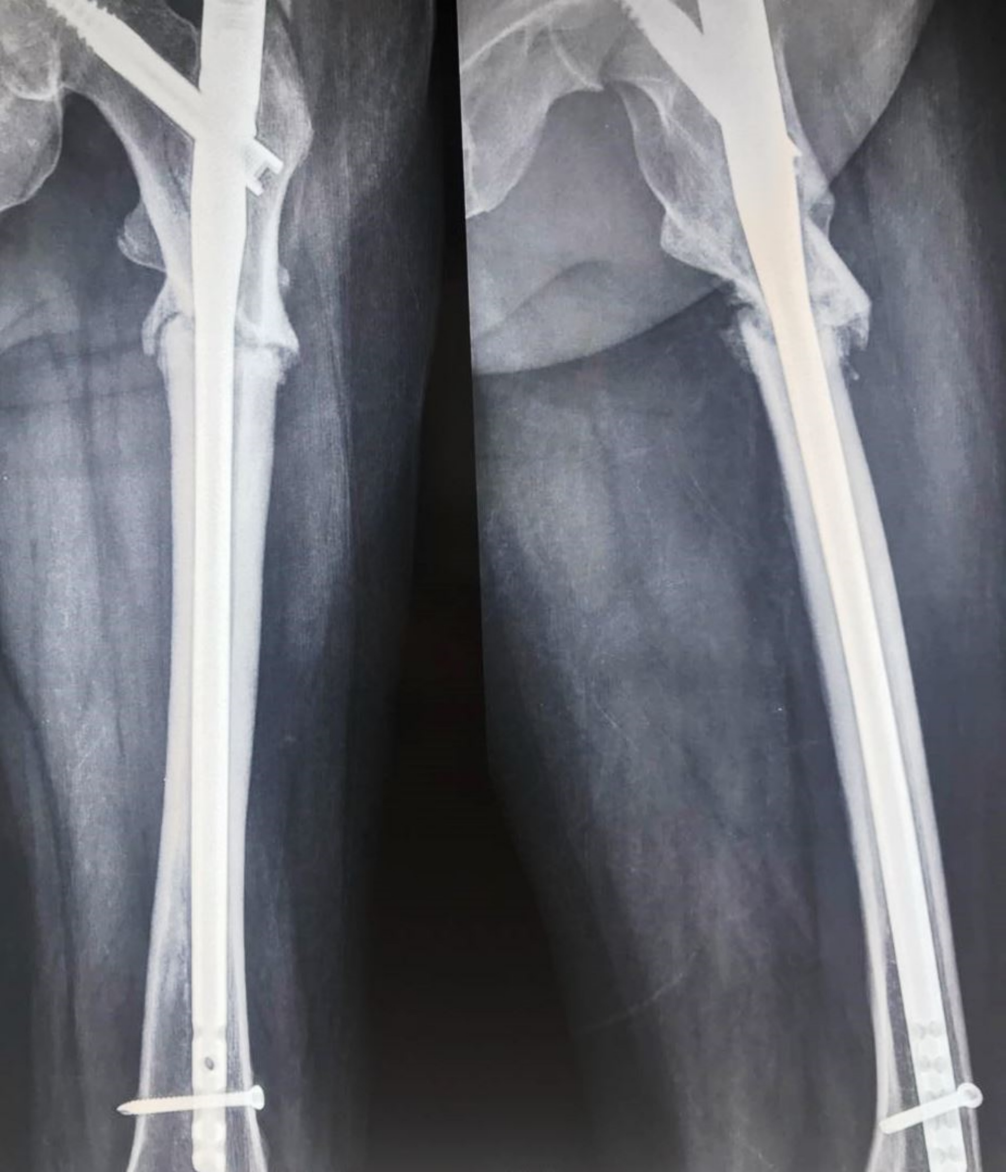
One-year postoperative X-ray showing nonunion at the fracture site.

**Figure 4:**
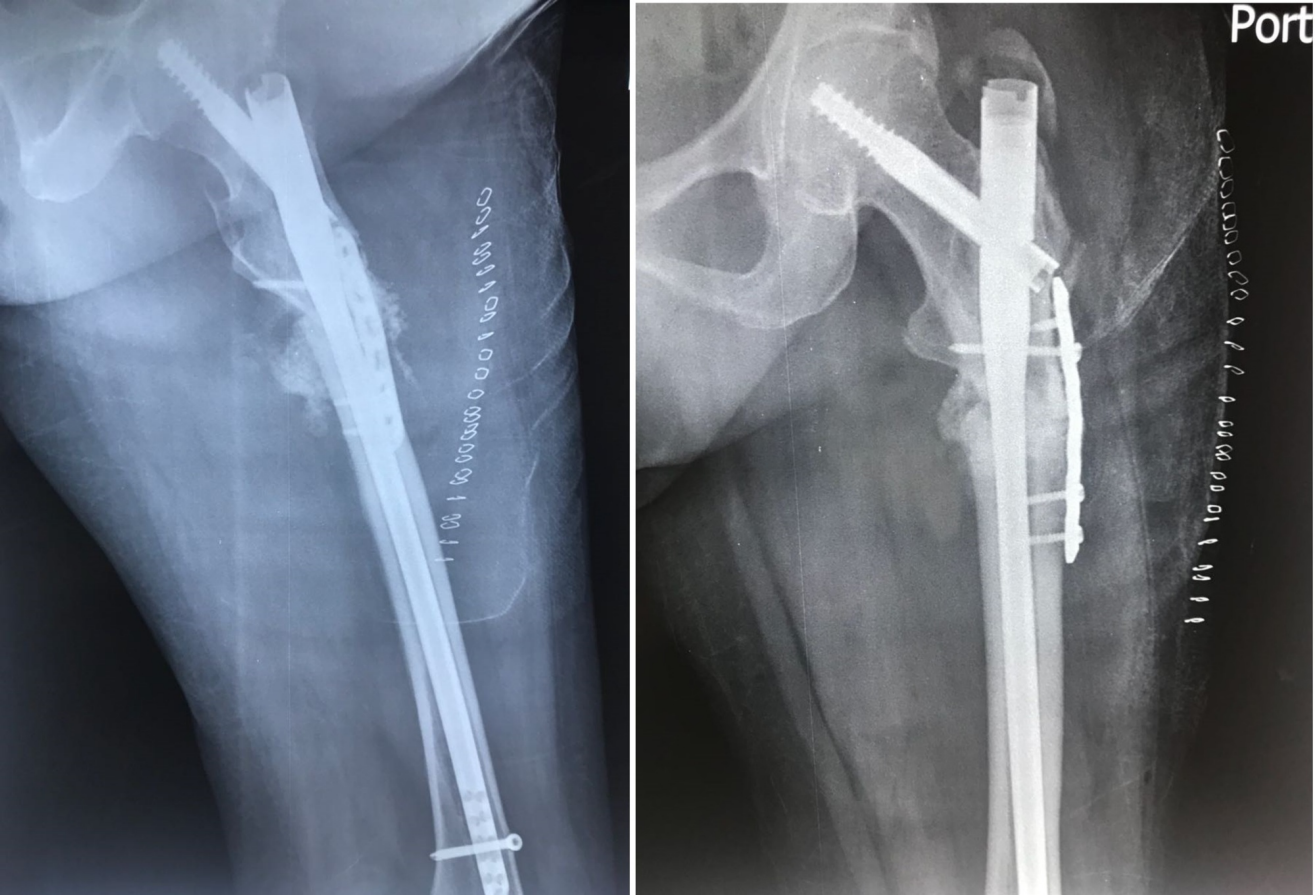
Immediate postoperative X-ray showing the augment plating done with bone grafting and bone marrow injection. Plating was done to provide compression at the fracture site.

On the subsequent X-rays, it was seen that the bone was not uniting; after nine months, the fracture was labeled as a nonunion subtrochanteric fracture (Figure 3). The patient was taken up for surgery after one year for the same. The fracture site was exposed and after freshening of the fracture ends it was seen that fixation was stable with intramedullary nail. However, additional stability and compression were achieved at the fracture site with a 6 hole 3.5 mm LC-DCP which was then fixed with four cortical screws inserted by “missing” technique. "Missing" technique entails the insertion of the plate screws so that they miss the the intramedullary nail. At the fracture site, a cortico-cancellous bone graft taken from the same side anterior superior iliac spine was impacted at the fracture site. About 60 ml of bone marrow was aspirated from the opposite iliac crest, and a 4 ml concentrate was prepared which was mixed in 10 cc granules of calcium triphosphate; the granules were placed all around the fracture site (Figure 4).

**Figure 5:**
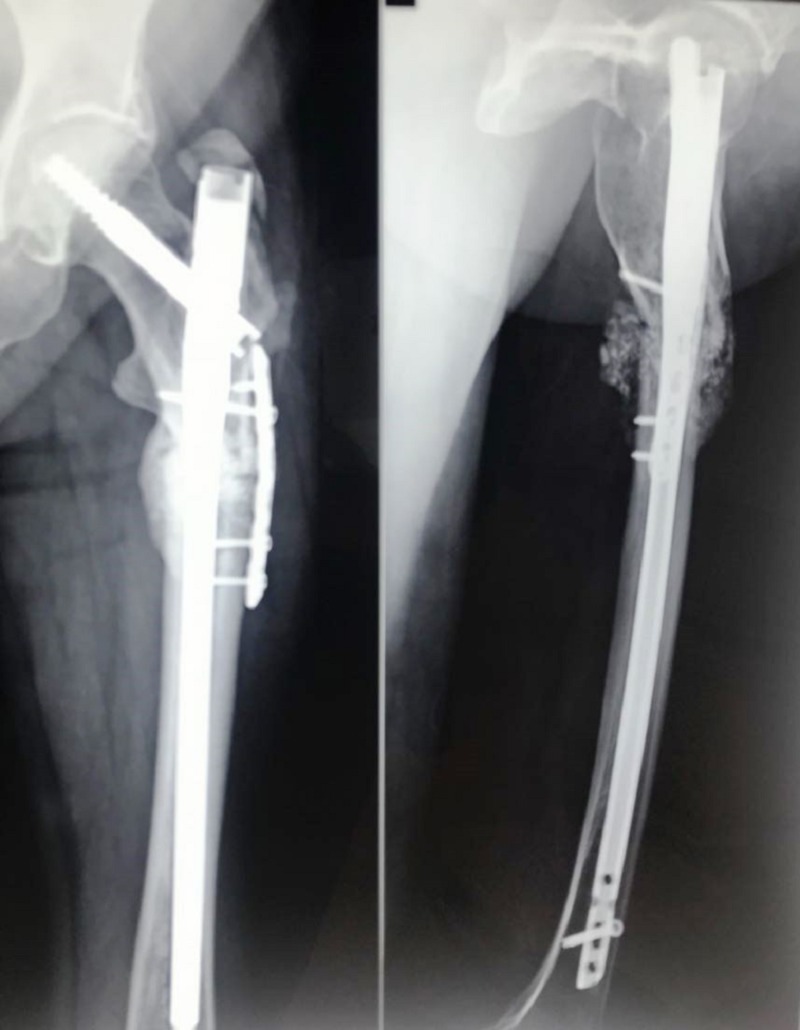
Three months postoperative showing signs of union at the fracture site.

Postoperative recovery was uneventful and the patient was mobilized using partial weight-bearing the next day. The fracture showed early signs of union, and three months postoperatively the fracture showed signs of complete union (Figure 5). Clinically the fracture also showed signs of union with the patient able to walk without any pain.

## Discussion

Bisphosphonates have been widely used for the management of bone loss and osteoporosis. Since 1976 when they were first used for the management of postmenopausal osteoporosis, they have shown wide usage and they have quickly become the primary drug for the management of osteoporosis [6]. Common side effects reported were gastric problems, osteonecrosis of the jaw, and in some cases even atrial fibrillation [7].

Odvina et al. were the first few authors to report the incidence of atraumatic nonspinal fractures in patients with long-term usage of bisphosphonates [7]. They reported such fractures in nine patients. Several cases have been reported ever since [5, 8]. Regarding the definition of atypical fractures, it was still unclear until Shane et al. came up with the criteria for the definition of atypical fractures [9]. These included fractures located between the lesser trochanter and supra­condylar flare, atraumatic or fractures with minimal trauma, a short oblique or transverse fracture configuration without communition, and a medial spike when fracture was complete. Minor criteria include prodromal symptoms, long duration of drug usage, and delayed healing. The fracture in this report was consistent with the above criteria and, hence, was labeled as an atypical fracture secondary to bisphosphonate usage. Latest recommendations regarding the long-term usage of bisphosphonates suggest the giving of a drug holiday which should be given after every three to 10 years of therapy for at least one to five years depending on the degree of osteoporosis and fragility fracture risk of the patient [10]. The patient in the study took alendronate regularly for five years without taking a break. 

The current recommendation for atypical fractures following long-term bisphosphonate therapy is fixation with intramedullary devices. In a review study by Koh et al. in 2017, it was concluded that the first line of management of atypical fractures following bisphosphonate use was fixation with intramedullary devices [1]. However, they have also mentioned that the fracture union might be delayed and that this union may be helped by using teriparatide and the immediate cessation of bisphosphonates. Conservative management of such fractures should not be attempted as surgical management may be required in around 50% of the patients. They have also recommended prophylactic nailing in cases of intractable pain on long-term usage of bisphosphonates.

In the above case study, the fracture was fixed with an intramedullary device initially. When the intramedullary device failed and the patient ended up with nonunion of the fracture, the patient underwent a secondary procedure and the fracture site was opened, freshened, and augmented with bone marrow concentrate and bone graft. The intramedullary nail was found to be stable in the intraoperative period and hence, the fracture was augmented with the help of plating. This led to the successful union of the fracture.

Initial failure of union might be attributed to the fact that bisphosphonates might still be present in the body up to five years after stopping the bisphosphonates. This is because bisphosphonates are absorbed in the bones, and when remodeling occurs, the bisphosphonates again get introduced into circulation, and are again absorbed by the bones. Hence, delayed union or nonunion may occur [10]. This may also explain the long holiday period recommended as a shorter holiday period would be ineffective.

## Conclusions

Atypical fractures following bisphosphonate use are on the rise due to increased usage of bisphosphonates for postmenopausal osteoporosis; however, this complication should be kept in mind and these drugs should not be started without specific indications. Intramedullary fixation of such fractures remains the first-line treatment of choice. However, the patient must be counseled regarding the incidence of delayed union and nonunion which may require secondary management.
